# Accelerated molecular breeding of a novel P/TGMS line with broad-spectrum resistance to rice blast and bacterial blight in two-line hybrid rice

**DOI:** 10.1186/s12284-018-0203-8

**Published:** 2018-02-17

**Authors:** Jiaming Mi, Dabing Yang, Yi Chen, Jiefeng Jiang, Haipeng Mou, Junbin Huang, Yidan Ouyang, Tongmin Mou

**Affiliations:** 1 National Key Laboratory of Crop Genetic Improvement and National Centre of Plant Gene Research (Wuhan), Huazhong Agricultural University, Wuhan, 430070 China; 2 College of Plant Science and Technology and the Key Lab of Crop Disease Monitoring and Safety Control in Hubei Province, Huazshong Agricultural University, Wuhan, 430070 China

**Keywords:** *Oryza sativa* L., Two-line hybrid rice, Blast resistance, Bacterial blight resistance, Marker-assisted selection, Next-generation sequencing

## Abstract

**Background:**

Breeding two-line hybrid rice with disease resistance is an effective approach to stabilize rice yield in commercial rice production of China.

**Results:**

We improved the blast and bacterial blight resistance of Guangzhan63-4S, an elite photoperiod- and thermo-sensitive male sterile (P/TGMS) line widely used in two-line hybrid rice, by introducing the *R* genes *Pi2* and *Xa7* conferring resistance to rice blast and bacterial blight, respectively. Through the backcrossing and gene pyramiding breeding coupled with molecular marker-assisted selection, a new P/TGMS line Hua1228S carrying *Pi2*, *Xa7*, and *tms5* was developed. Based on 200,000 SNP markers by next-generation sequencing, Hua1228S covered 87.6% of the recurrent genome, as well as 4.5% of the donor genome from VE6219 and 7.9% from YR7029–39. When infected with seven tested *Xanthomonas oryzae* pv. *oryzae* strains, Hua1228S conferred high resistance (0 level) to six bacterial blight strains. Moreover, Hua1228S showed broad-spectrum resistance to rice blast isolates with a high resistance frequency of 90.91%. High levels of resistance to leaf blast and neck blast were observed under heavy disease pressure in natural field. Importantly, Hua1228S showed identical fertility-sterility alteration pattern to Guangzhan63-4S. Thus, two hybrid combinations Hua Liangyou 2821 and Hua Liangyou 284 derived from Hua1228S exhibited enhanced resistance and higher yield compared with the control variety Feng Liangyou 4.

**Conclusions:**

These results indicate that Hua1228S has tremendous potentiality to increase and stabilize the rice yield, through the introgression of two *R* genes by marker-assisted selection strategy.

**Electronic supplementary material:**

The online version of this article (10.1186/s12284-018-0203-8) contains supplementary material, which is available to authorized users.

## Background

Hybrid rice (*Oryza sativa* L.) has provided a fundamental guarantee for food supply all over the world (Cheng et al. 2007). It has contributed 65% of the total rice production accounting for 57% of the total rice planting area in China (Yuan 2014). Compared with the three-line hybrid rice, the two-line hybrid rice has played an increasingly significant role in rice production due to the avoidance of negative effects by sterility-inducing cytoplasm and its independence on the restorer genes. In the past decade, breeders have paid more attention to super two-line hybrid rice for increasing yields (Yuan 2017). During the past decade, the proportion of planting area for two-line hybrid rice has significantly increased by 25% in China (Mou 2016; Ni et al. 2015).

Stable sterility period was crucial for commercial thermo-sensitive genic male sterility (TGMS) and photoperiod-sensitive genic male sterility (PGMS) lines in a given region, and is also the reliable safety guarantee for the two-line hybrid rice seed production in China (Jiang et al. 2015). The P/TGMS trait is governed by major genes, thus enabling their easy transfer into any genetic background and increasing genetic diversity of the P/TGMS lines (Mou 2016). To date, two PGMS genes (*Pms1* and *pms3*) from PGMS line Nongken58S and one TGMS gene (*tms5*) from TGMS line Annong-1S have been cloned. Compared with the completely recessive loci *pms3* and *tms5*, male sterility conditioned by *Pms1* is semi-dominant. Coincidentally, the functional mutations of *Pms1*, *pms3* and *tms5* are all SNP variations (Ding et al. 2012; Fan et al. 2016; Zhou et al. 2012; Zhou et al. 2014). It is interesting to note that these findings would facilitate marker-assisted selection of P/TGMS trait in process of genetic improvement for two-line hybrid rice.

Rice growth and yield are frequently and severely affected by the most devastating diseases, rice blast and bacterial blight caused by *Pyricularia grisea* and *Xanthomonas oryzae* pv. *oryzae* (*Xoo*), respectively (Cheng et al. 2007; Zhang 2007). Therefore, enhancing rice blast and bacterial blight resistance of two-line hybrids was considered as the most cost-effective and eco-friendly approach to increase yield (Zhang 2007). Relative to the conventional method of chemical pesticides with additional costs in rice production and chemical contamination of environment and food, the application of disease resistance (*R*) genes is considered as an optimal strategy to minimize the yield loss caused by diseases.

A total of 102 *R* genes conferring rice blast resistance have been identified (Xiao et al. 2016). Many broad-spectrum allelic genes, such as *Pi2*, *Pi9*, *Pigm*, *Pi50* and *Piz-t* on chromosome 6, have been validated and each allelic gene controls resistance to a distinct set of *M. oryzae* isolates (Deng et al. 2017; Jiang et al. 2012; Qu et al. 2006; Su et al. 2015; Zhou et al. 2006). In addition, forty *R* genes conferring bacteria blight resistance have been identified and are designated as *Xa1* to *Xa40* (Kim et al. 2015). Among them, *Xa21* and *Xa23* on chromosome 11, and *Xa7* on chromosome 6 exhibit the broadest resistance spectrum and the strongest resistance to bacterial blight (Zhang 2009). Hence, it is essential to incorporate these valuable *Pi*- and *Xa*- genes into commercial elite lines, which will extend disease resistance longevity in released varieties. Recently through application of marker-assisted backcrossing, many *R* genes for blast and bacterial blight have been successfully introgressed into elite inbred lines of rice to improve the disease resistance. Some new inbred varieties that combine excellent grain quality with resistance to diseases and input use efficiency have been popularized in Southeast Asia (Joseph et al. 2004; Luo et al. 2014; Ratna Madhavi et al. 2016; Tanweer et al. 2015; Xiao et al. 2016).

Similarly, marker-assisted selection has also been implemented in the improvement of parental lines of hybrid rice for resistance to bacterial blight and blast diseases. Incorporation of *R* genes into the elite restorer line (paternal line) has been recognized as an efficient way to resist the diseases in hybrid rice (Chen et al. 2001; Fan et al. 2017; Gouda et al. 2013; Luo et al. 2012; Singh et al. 2013; Xiao et al. 2016; Zhang et al. 2006). However, in F_1_ hybrid rice, the resistance to disease may also be influenced by the incomplete dominance of *R* genes in the heterozygous background (Luo et al. 2012; Zhang et al. 2006). Thus, transferring the *R* genes into the male sterile line (maternal parent) is critical for further enhancing disease resistance of hybrid rice. So far, little attention has been paid to the improvement of male sterile line for disease resistance through marker-assisted selection. The lack of adequate research is due to the challenge in reconstituting the P/TGMS traits, such as stable sterility period. Nevertheless, maintenance of P/TGMS traits while incorporating the resistance genes into P/TGMS line has been successfully demonstrated in two-line hybrids rice breeding (Jiang et al. 2015; Ni et al. 2015).

Guangzhan63-4S is a widely used female parent for two-line hybrid rice breeding in China. The male sterility of Guangzhan63-4S is determined by both temperature and day length, which is designated as a photoperiod- and thermo-sensitive male sterility line. In particular, more than twenty-four hybrid combinations derived from Guangzhan63-4S have been released in the middle-lower Yangzi River basin region of China, due to their high yield potential and excellent grain quality (http://www.ricedata.cn/variety/varis/601234.htm). However, these hybrids are susceptible to rice blast and bacterial blight, which are potential threats to the rice production. In this study, two broad-spectrum durable *R* genes, *Pi2* and *Xa7* that located on chromosome 6, were introduced into Guangzhan63-4S through molecular marker-assisted pyramiding and phenotypic selection (Chen et al. 2008; Jiang et al. 2012). A novel photoperiod- and thermo-sensitive male sterility line was developed with improved disease resistance to both blast and bacterial blight.

## Result

### Development of Hua1228S

**Fig. 1:**
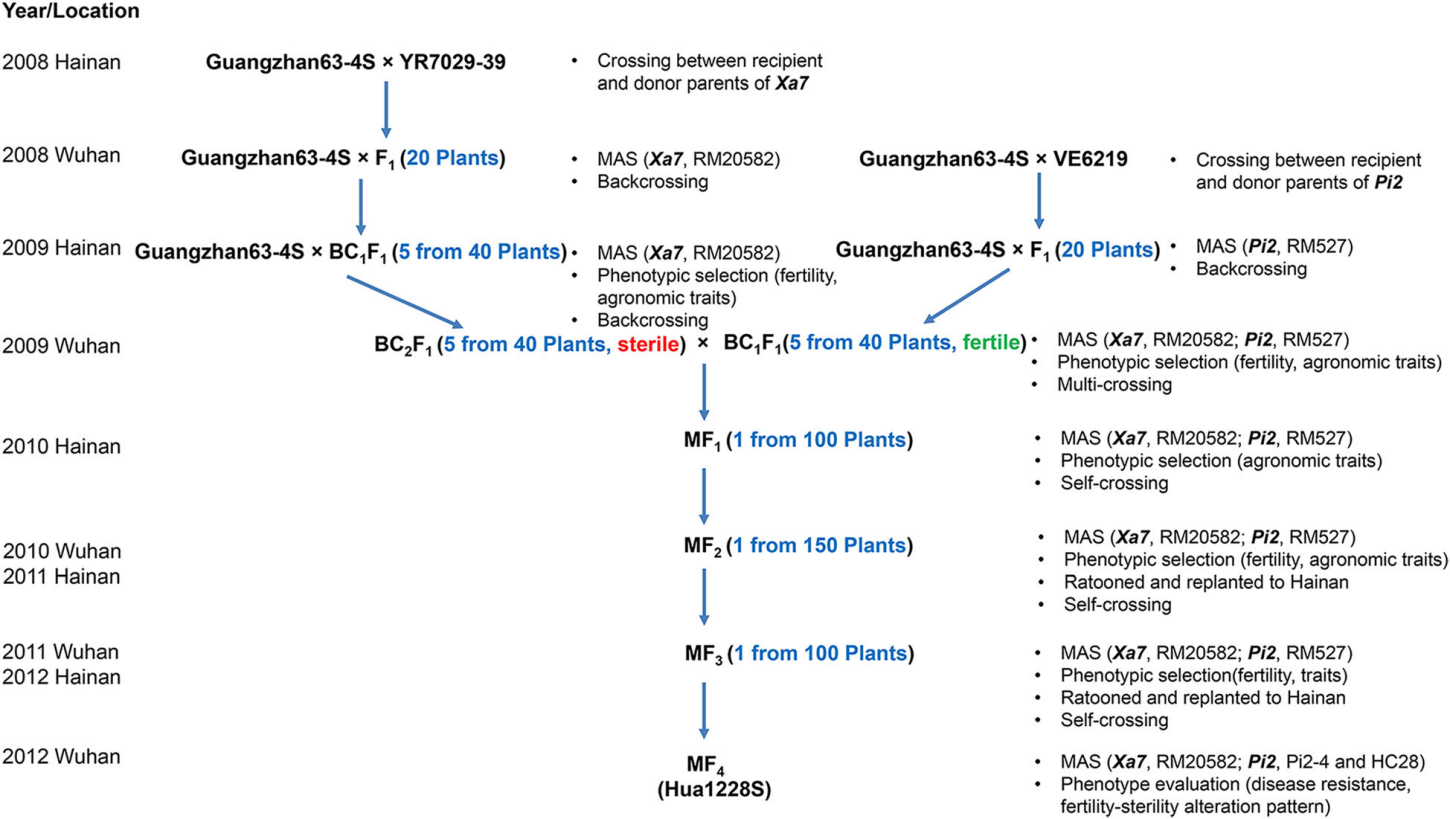
The flowchart for the development of disease-resistant Hua1228S. MAS, marker-assisted selection

In this study, two disease resistance genes of *Xa7* and *Pi2* were transferred into an elite P/TGMS line Guangzhan63-4S. As shown in the schematic flow (Fig. 1), two donor lines YR7029–39 and VE6219 harboring homozygous *Xa7* and *Pi2* genes, respectively, were used to backcross with the recurrent female parent Guangzhan63-4S. In the backcross generation, forty BC_2_F_1_ plants of Guangzhan63-4S/YR7029–39 and forty BC_1_F_1_ plants of Guangzhan63-4S/VE6219, harboring heterozygous *Xa7* and *Pi2* genes, respectively, were obtained. Two sets of intermediate materials showed segregation in pollen fertility under long day and high temperature conditions at Wuhan in summer season of 2009. To pyramid *Xa7*, *Pi2*, and P/TGMS genes, five male sterile individuals with *Xa7* from BC_2_F_1_ of Guangzhan63-4S/YR7029–39 were selected as the female recipients, while five fertile individuals with *Pi2* from BC_1_F_1_ of Guangzhan63-4S/VE6219 were selected as the male parent by mixing pollen grains. Subsequently, the intercross of Guangzhan63-4S/YR7029–39//Guangzhan63-4S/VE6219 was made, and the commingling seeds derived from five male sterile plants were collected to generate the MF_1_. Among 100 plants in MF_1_, twenty-one individuals with heterozygous *Xa7* and *Pi2* were found by PCR analysis, and one plant with similar morphological phenotypes to that of Guangzhan63-4S was selected for self-pollination under short day and low temperature conditions at Hainan in winter-spring season of 2009–2010. In MF_2_, among the 150 male sterile plants, 66 plants containing *Xa7* and *Pi2* were identified. One sterile MF_2_ individual almost identical to Guangzhan63-4S but with heterozygous *Xa7* and homozygous *Pi2* was selected and ratooned at Wuhan in summer season of 2010, and then the stubbles were replanted to generate MF_3_ by self-pollination at Hainan. Consequently, 18 plants homozygous for the double-genes, *Xa7* plus *Pi2*, were obtained in MF_3_ and they exhibited little morphological segregation and high similarity to Guangzhan63-4S. Finally, one plant with the best trait performance was selected and self-pollinated in Hainan to generate MF_4_. The MF_4_ plants in Wuhan exhibit stable morphology like that of Guangzhan63-4S, and therefore designated as a new P/TGMS line called Hua1228S.

### Background profiling by next generation sequencing

**Fig. 2:**
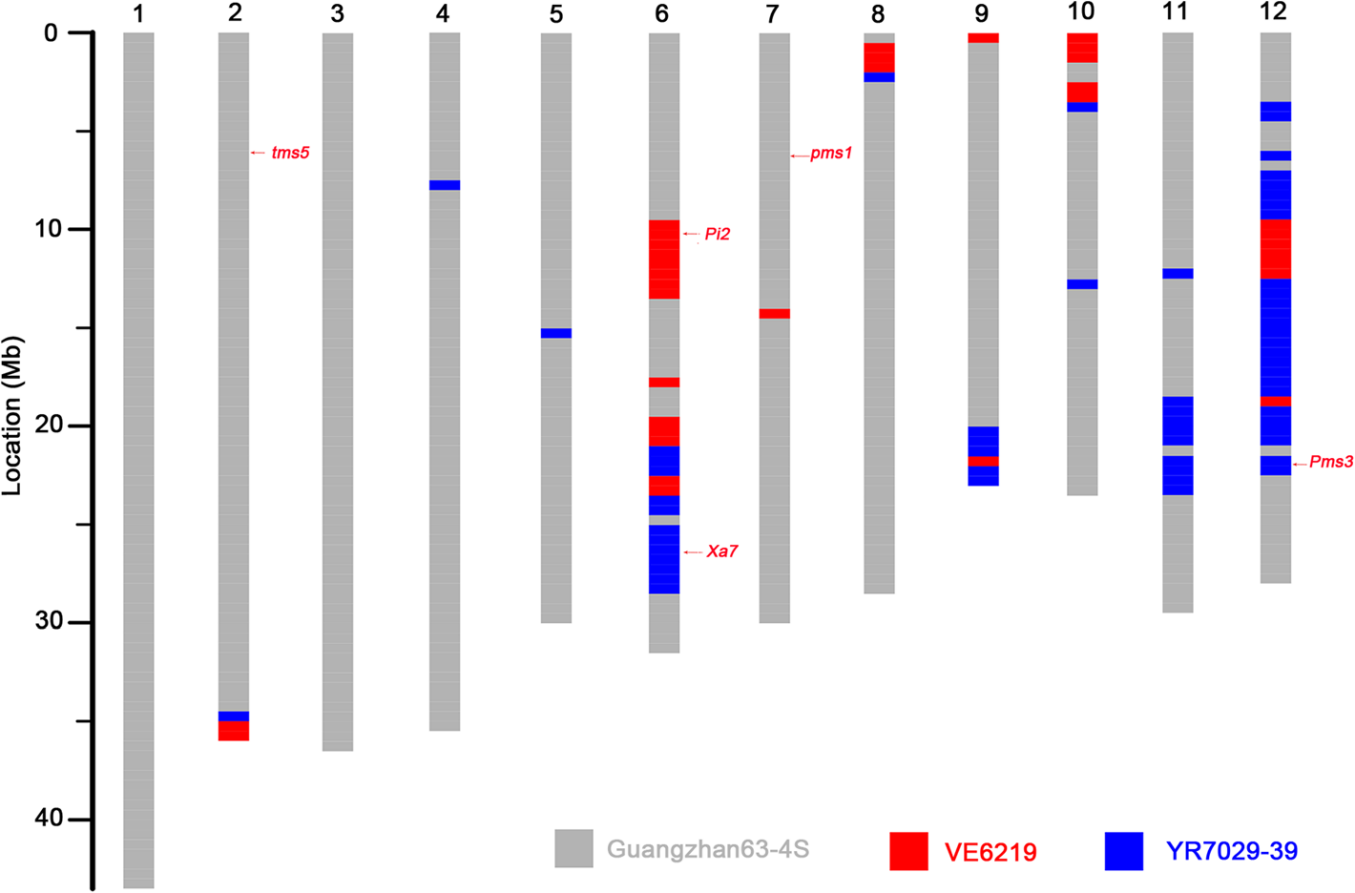
Genotype of the newly-developed P/TGMS line Hua1228S with disease resistance under the generic background of Guangzhan63-4S, based on the next-generation sequencing. Twelve chromosomes of rice are labeled with 1 to 12. The reference genome is Nipponbare (MSU 7.0). The gray, red, and blue boxes indicate the substituted segments of the recurrent parent Guangzhan63-4S and the two donor parents VE6219 and YR7029–39, respectively

Sequencing data of Hua1228S, and three breeding stock lines Guangzhan63-4S, VE6219 and YR7029–39, were generated by Illumina HiSeq PE150 platform in the form of 150-bp paired-end reads. In total, we achieved an effective depth of 10 × coverage for each line, and identified 200,000 high-confidence SNPs distributing evenly in the whole genome. This collection of SNPs provided high-density marker coverage with the average density of 1 SNP per 2-kb. In order to assess the genetic background of Hua1228S, we scanned the genome of Hua1228S and set different colors for genomic regions according to the type of haplotype block (Fig. 2). The results showed that Hua1228S had twenty-one substituted segments from VE6219 or YR7029–39 distributing on 10 chromosomes, respectively. Maximum donor parent segments were present in chromosome 12 possibly due to positive selection of desirable traits from the donor parents. The analysis in genetic background revealed that Hua1228S covered up to 87.6% of the recurrent parent genome (Guangzhan63-4S), while 4.5% and 7.9% of genomes from the donors VE6219 and YR7029–39, respectively (Fig. 2). Hence, we conclude that high-resolution genome scans based on next-generation sequencing can be used to identify genomic component from multiple breeding parents.

### Fertility-sterility alteration pattern in Hua1228S determined by *tms5*

**Fig. 3:**
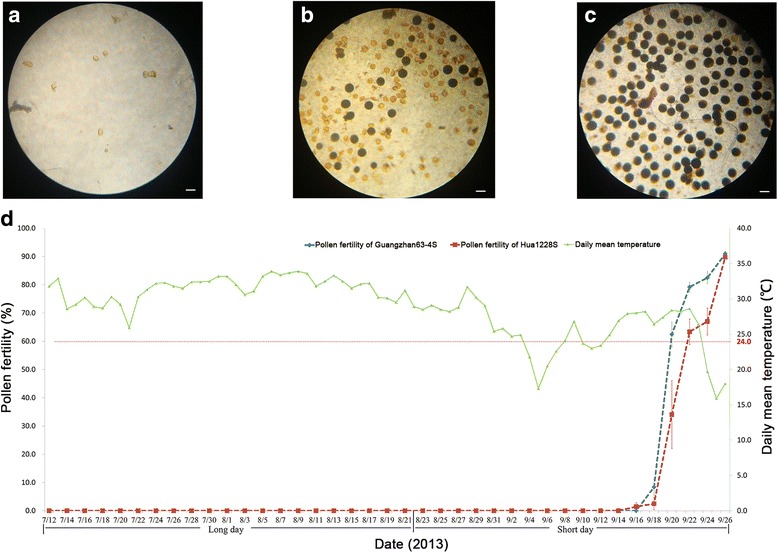
Fertility-sterility alteration pattern of Hua1228S in 2013 at Wuhan. **a**-**c** Pollen grains from Hua1228S on 15th August (**a**), 18th September (**b**) and 26th September (**c**). **d** Dynamic pollen fertility of the newly-developed P/TGMS lines Hua1228S and Guangzhan63-4S relative to daily mean temperature and day length from 12th July to 26th September. Error bars, s. e. m. Scale bars: 50 μm

P/TGMS lines are male-sterile under restrictive conditions of long day and high temperatures, but converted to male-fertile and self-pollination under permissive conditions of short day and low temperatures. In this study, Hua1228S was completely male-sterile with typically abortive pollen grains or no pollen until mid-September at Wuhan, which is identical to the recurrent parent Guangzhan63-4S (Fig. 3a and d). When the daily mean temperature declined below 24 °C with the day-length shorter than 13.5 h during 4–12 September, partial fertility with the pollen fertility of 8.4% and 2.5% was generated in Guangzhan63-4S and Hua1228S on 18 September (Fig. 3b and d). With the temperature dropping later, the pollen fertility of two lines finally convert to 91.0% and 89.8% on 26 September (Fig. 3c and d), which showed a high fertility restoration efficiency. These results indicated that the newly-developed P/TGMS line Hua1228S is suitable for the practical application in seed production of two-line hybrid rice.

We further identified the corresponding mutated gene related to male sterility in both Hua1228S and Guangzhan63-4S by sequencing the cloned loci *PMS1*, *PMS3* and *TMS5* conferring P/TGMS trait. Guangzhan63-4S carried the *tms5* mutation, but not the *Pms1* and *pms3* mutations (Fig. 2; Ding et al., 2012; Zhou et al., 2014; Fan et al., 2017). The newly-developed P/TGMS line Hua1228S had also harbored *tms5* mutation (Additional file 1: Table S2). In addition, the fragment of *Pms3* in Hua1228S was derived from the donor YR6029–39 with normal fertility, implying *pms3* is not the causal genes of P/TGMS trait of Hua1228S (Fig. 2). These results demonstrated that the loss-of-function mutation of *TMS5* confers P/TGMS trait in Hua1228 and its recurrent parent Guanzhan63-4S. Therefore, we conclude that the desired gene *tms5* was successfully reserved through phenotypic selection.

### Hua1228S showing broad-spectrum resistance to rice blast and bacterial blight

**Fig. 4:**
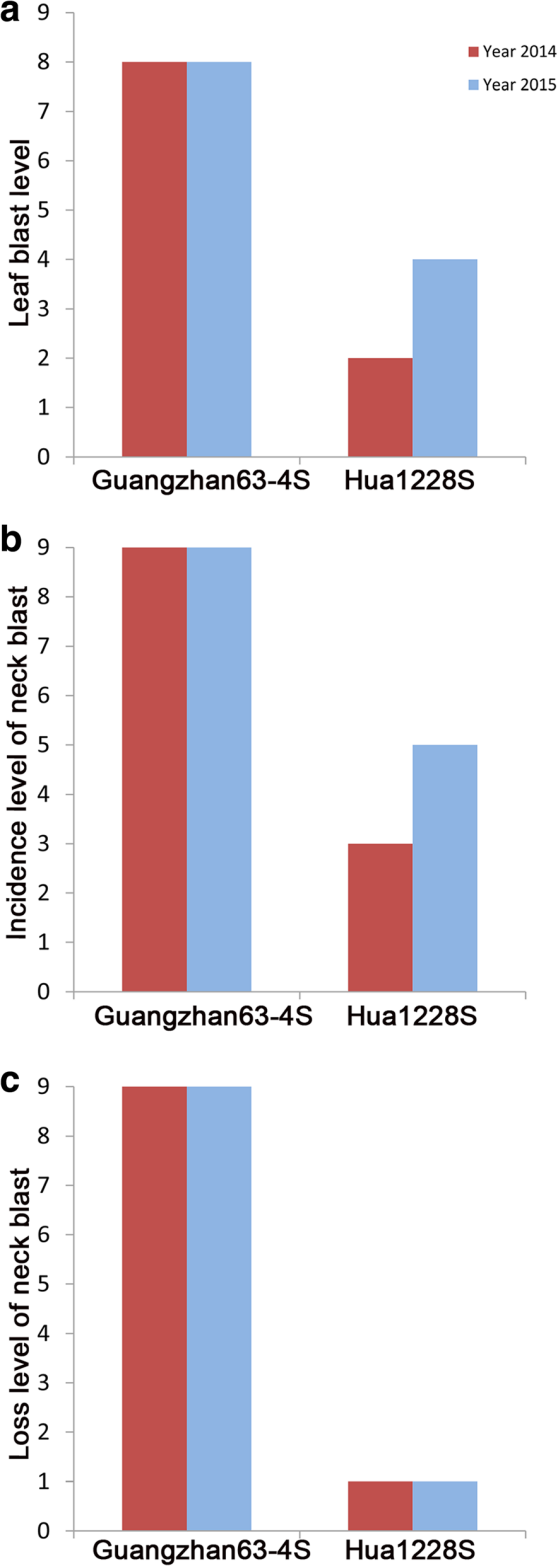
Blast resistance level of Hua1228S and Guangzhan63-4S in natural field with serious blast epidemic in 2014 and 2015. **a** Leaf blast resistance level at tillering stage. **b** Incidence level of neck blast at maturity stage. **c** Loss level of neck blast at maturity stage

To test whether Hua1228S can improve the blast resistance, we evaluated the blast resistance of Hua1228S in the natural field in two rice-growing seasons of 2014 and 2015. Both the leaf and neck blast resistance of Hua1228S were evaluated, using the recurrent parent Guangzhan63-4S as the control. In both of the two years, Guangzhan63-4S showed 8 level of leaf blast, 9 level of neck blast incidence, and 9 level of neck blast loss under heavy blast disease pressure, being highly susceptible to rice blast. Hua1228S with the *Pi2* gene exhibited different resistance performance between the two years. In average, Hua1228S expressed moderate resistance to leaf blast (3 level) and neck blast incidence (4 level), and resistance to neck blast loss (1 level) (Fig. 4). In addition, blast resistance spectrum of Hua1228S was assessed with 33 blast isolates under greenhouse conditions, using Guangzhan63-4S and the blast susceptible variety CO39 as the control. Hua1228S showed a high resistance frequency covering 90.91% of the rice blast isolates, while Guangzhan63-4S was resistant to four isolates accounting for only 12.12% of the total (Table 1). These results suggested an enhanced and broad-spectrum resistance to blast has been obtained for Hua1228S.

**Table 1:** Disease reaction of Hua1228S to 33 isolates of *M. oryzae*

Blast race	Isolates No.	CO39 (CK)	Guangzhan63-4S	Hua1228S
ZA01	GD-15KP01	S	S	R
ZA01	GD-15KP02	S	S	R
ZA05	GD-15KP03	S	S	R
ZA13	GD-15KP04	S	S	R
ZA13	GD-15KP05	S	S	R
ZA13	GD-15KP06	S	S	R
ZA13	GD-15KP07	S	S	R
ZA13	GD-15KP08	S	S	S
ZA15	GD-15KP09	S	R	R
ZA37	GD-15KP10	S	S	R
ZB01	GD-15KP11	S	S	R
ZB01	GD-15KP12	S	S	R
ZB05	GD-15KP13	S	S	R
ZB13	GD-15KP14	S	S	R
ZB13	GD-15KP15	S	S	R
ZB13	GD-15KP16	S	S	R
ZB13	GD-15KP17	S	S	R
ZB13	GD-15KP18	S	S	R
ZB13	GD-15KP19	S	S	R
ZB13	GD-15KP20	S	S	R
ZB13	GD-15KP21	S	S	R
ZB13	GD-15KP22	S	S	R
ZB13	GD-15KP23	S	S	S
ZB13	GD-15KP24	S	S	R
ZB13	GD-15KP25	R	S	S
ZB13	GD-15KP26	S	S	R
ZC03	GD-15KP27	S	R	R
ZC05	GD-15KP28	S	R	R
ZC13	GD-15KP29	S	S	R
ZC13	GD-15KP30	S	S	R
ZC13	GD-15KP31	S	S	R
ZC13	GD-15KP32	S	S	R
ZC15	GD-15KP33	S	R	R
Amount of incompatible isolates		1	4	30
Percentage of incompatible isolates (%)		3.03	12.12	90.91

Note: R and S indicate resistance and susceptibility, respectively

**Fig. 5:**
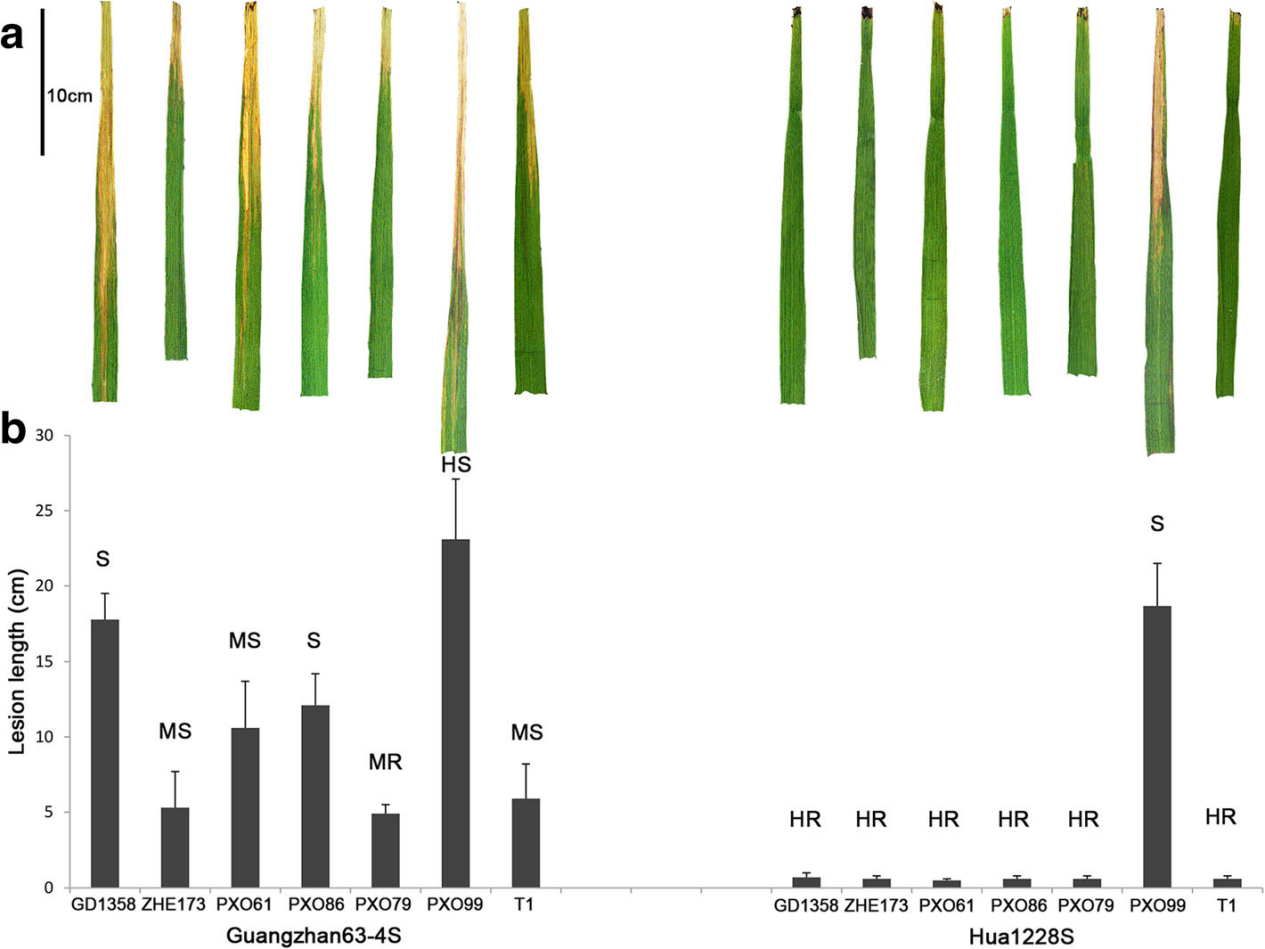
Reaction patterns of Hua1228S and Guangzhan63-4S to seven *Xoo* strains. **a** Lesion patterns on leaves of Guangzhan63-4S and Hua1228S plants were used to show the lesion patterns. Pictures were taken at 21st-day after inoculation. **b** Resistance level of bacterial blight based on the lesion length. Lesion length was scored at 21st-day after inoculation. HR, high resistance; R, resistance; MR, moderate resistance; MS, moderate susceptible; S susceptible; HS, high susceptible. Scale bars: 10 cm. Error bars, s. d

For the evaluation of bacterial blight resistance, seven *Xoo* strains were used to inoculate Guangzhan63-4S and Hua1228S. Bacterial blight resistance of Hua1228S to seven *Xoo* strains was significantly higher (*P* < 0.01), compared to that of Guangzhan63-4S. Hua1228S conferred high resistance to six of the seven *Xoo* strains with lesion lengths ranging from 0.5 to 0.7 cm. By contrast, the recipient parent Guangzhan63-4S was susceptible to six *Xoo* strains with longer lesion lengths ranging from 5.3 to 32.1 cm. Hua1228S was still susceptible to PXO99, a prevalent strain in the Philippines, with a lesion length of 18.7 cm, whereas Guangzhan63-4S was highly susceptible to PXO99 with a longer lesion length of 23.1 cm (Fig. 5). These results indicated a high and broad-spectrum bacterial blight resistance of Hua1228S. In particular, Hua1228S was highly resistant against the two epidemic prevalent *Xoo* strains, ZHE173 and GD1358, in rice growing area of southern China.

### Evaluation of agronomic performance of Hua1228S

To compare the agronomic performance between Hua1228S and Guangzhan63-4S, nine agronomic traits, including days to heading, plant height, panicle number, panicle length, number of spikelet per panicle, seed setting rate, 1000-grain weight, yield per plant, and stigma exertion rate, were investigated in both Wuhan and Hainan under no disease stress (Table 2). In the summer season of 2014 at Wuhan, Hua1228S did not show any significant difference from Guangzhan63-4S for all of the agronomic traits. However, in the winter season of 2015 at Hainan, the plant height, panicle length and 1000-grain weight of Hua1228S had a significant increase in comparison with the recurrent parent Guangzhan63-4S. Relative to Guangzhan63-4S, the plant height of Hua1228S was 6.6 cm higher, with an average 0.9 cm longer panicle length. In addition, Hua1228S possessed heavier 1000-grain weight by 1.1 g than that of Guangzhan63-4S (Table 2). These differences might be caused by the introgression of fragment from the donor YR7029–39 or VE6219 (Fig. 2). Nevertheless, no significant difference was observed in terms of yield per plant in the winter season of 2015 at Hainan (Table 2). Thus, the reproductive capacity of Hua1228S under permissive conditions of short day and low temperatures are on par with the recurrent parent Guangzhan63-4S in seed multiplication.

**Table 2:** Agronomic performance of the improved line Hua1228S and the recurrent parent Guangzhan63-4S in Wuhan and Hainan

Trait	Summer season (sterile) at Wuhan in 2014		Winter season (fertile) at Hainan in 2015	
	Guangzhan63-4S	Hua1228S	Guangzhan63-4S	Hua1228S
Days to heading (d)	87.3 ± 6.1	88.0 ± 6.1	108.0 ± 9.5	108.7 ± 9.1
Plant height (cm)	91.6 ± 4.7	93.0 ± 4.6	94.7 ± 2.0	101.3 ± 2.5^**^
Panicle number	9.9 ± 1.3	9.4 ± 2.0	7.1 ± 1.3	7.1 ± 0.9
Panicle length (cm)	24.5 ± 1.0	24.1 ± 1.5	21.3 ± 0.4	22.2 ± 0.7^*^
Number of spikelet per panicle	158.9 ± 13.5	161.4 ± 14.1	156.3 ± 5.3	162.2 ± 4.3
Seed setting rate (%)	**–**	**–**	84.8 ± 5.1	82.7 ± 4.0
1000-grain weight (g)	**–**	**–**	27.8 ± 0.6	28.9 ± 1.0^*^
Stigma exertion rate (%)	35.9 ± 15.5	53.7 ± 9.2	**–**	**–**
Yield per plant (g)	**–**	**–**	25.8 ± 3.5	27.4 ± 4.2

Note: The value of agronomic traits is measured as mean ± s. d. ** and *indicate significant difference of the trait performance between the improved line and the control line at *P* < 0.01 and *P* < 0.05, respectively

### Evaluation of derived hybrids

Two hybrid combinations derived from Hua1228S, Hua1228S/Huimin121 (named as Hua Liangyou 2821) and Hua1228S/Yandao4 (named as Hua Liangyou 284), were evaluated in the Hubei and National Rice Region Trials, respectively, in both 2015 and 2016. Data on blast and bacterial blight resistance and yield of the two hybrids are presented in Table 3. Enhanced blast and bacterial blight resistance were obtained in the hybrid combinations Hua Liangyou 2821 and Hua Liangyou 284, compared with the control variety Feng Liangyou 4. Hua Liangyou 2821 carrying the heterozygous *Pi2* gene showed moderate resistance (3.3) to leaf blast, moderate susceptibility (5.0 in 2015, 4.5 in 2016) for neck blast incidence, and resistance (1.5) for neck blast loss, respectively. In comparison with Hua Liangyou 2821, Hua Liangyou 284 exhibited relatively lower resistance with moderate resistance (2.7 in 2015, 3.3 in 2016) to leaf blast, susceptibility (6.7 in 2015, 7.7 in 2016) for neck blast incidence, and moderate resistance (3.7 in 2015, 3.9 in 2016) for neck blast loss. Additionally, Hua Liangyou 2821 and Hua Liangyou 284, carrying the heterozygous *Xa7* gene, conferred resistance or moderate resistance to *Xoo* isolate ZHE173 with the lesion length ranging from 1.3 to 4.7 cm. Apart from the disease resistance advantage, the higher yield was observed in the hybrid combinations Hua Liangyou 2821 and Hua Liangyou 284, relative to the control variety Feng Liangyou 4 (Table 3). These results indicated that pyramiding *Pi2* and *Xa7* genes in Hua1228S could improve blast and bacterial blight resistance and broaden the resistance spectrum of two-line hybrid rice.

**Table 3:** Blast/bacterial blight resistance and yield test for the hybrid combinations Hua Liangyou 2821 and Hua Liangyou 284 in 2015 and 2016

Trait	Hubei Rice Variety Regional Trials^a^		National Rice Variety Regional Trials^b^	
	Hua Liangyou 2821	Feng Liangyou 4 (CK)	Hua Liangyou 284	Feng Liangyou 4 (CK)
2015				
Leaf blast score	3.3 ± 1.0^**^	6.8 ± 1.3	2.7 ± 1.8^**^	5.5 ± 2.2
Incidence score of neck blast	5.0 ± 1.6	8.0 ± 2.0	6.7 ± 0.8^*^	8.7 ± 0.8
Loss score of neck blast	1.5 ± 1.0^*^	7.0 ± 2.8	3.7 ± 1.0^**^	7.7 ± 1.6
Lesion length of bacterial blight (cm) ^c^	4.2 ± 0.9^**^	10.3 ± 1.1	1.3 ± 0.2^**^	8.6 ± 1.7
Yield (t ha^−1^)	10.41 ± 1.06^*^	10.11 ± 1.13	9.97 ± 1.25^**^	9.42 ± 1.29
2016				
Leaf blast score	3.3 ± 1.5^*^	6.8 ± 1.3	3.3 ± 2.2^*^	6.7 ± 1.5
Incidence score of neck blast	4.5 ± 1.9^*^	8.5 ± 1.0	7.7 ± 2.4	8.7 ± 0.8
Loss score of neck blast	1.5 ± 1.0^*^	7.0 ± 2.8	3.9 ± 2.1^*^	7.7 ± 1.0
Lesion length of bacterial blight (cm)^c^	2.0 ± 0.2^**^	7.1 ± 0.7	4.7 ± 1.2^**^	11.1 ± 1.3
Yield (t ha^− 1^)	8.97 ± 1.35	9.04 ± 1.42	9.33 ± 1.27^*^	8.88 ± 0.93

Note: ^a^Hubei Rice Variety Regional Trials were conducted in 9 field test sites according to methods and standards of Rice Variety Regional Trial in Hubei province. ^b^National Rice Variety Regional Trials were conducted in 21 field test sites according to methods and standards of National Rice Variety Regional Trial. ^c^ indicates the lesion length to *Xoo* isolate ZHE173. The value of traits is measured as mean ± s. d. ** and * indicate significant difference of the trait performance between the improved hybrid combinations and the control variety Feng Liangyou 4 at *P* < 0.01 and *P* < 0.05, respectively

## Discussion and conclusions

Breeding disease-resistant P/TGMS lines would facilitate the utilization of heterosis in rice. Using an integrated molecular breeding strategy with one or two generations of backcrossing followed by four generations of pedigree selection, we successfully transferred two disease resistance genes, *Xa7* and *Pi2*, into an elite P/TGMS line Guangzhan63-4S, to develop a new P/TGMS line Hua1228S. Pyramiding of *Xa7* and *Pi2* in Hua1228S had significantly increased the bacteria blight and blast resistance, without losing desirable traits of Guangzhan63-4S.

In the past decades, major *R* genes, including *Xa7* and *Pi2*, have been frequently introgressed or pyramided into modern adapted or elite rice genetic backgrounds from exotic germplasm via marker-assist backcrossing (Huang et al. 2012; Jiang et al. 2012). These novel germplasm accessions with excellent traits are suitable for parents of breeding populations in future rice improvement. This allows breeders to focus attention on a few of target traits in each generation. Moreover, it will greatly reduce the selection population size and the generations for backcrossing to get rid of undesired traits in the breeding program. Therefore, we used two modern improved lines YR7029–39 and VE6219 as donor parents to consecutively improve the bacterial blight and blast resistance of the P/TGMS lines. The result demonstrated that rational use of donor parents and appropriate backcrossing facilitated us to successfully develop a new P/TGMS line Hua1228S with bacterial blight, blast resistance and P/TGMS trait within only five years.

Compared to the inbred lines, the *Xa7* gene-mediated resistance to bacteria blight is decreased by the incomplete dominance of *Xa7* gene in the heterozygous background of the hybrid rice (Zhang et al. 2006). By introducing *Xa7* to male sterility line (maternal parent) and restorer line (paternal parent) simultaneously, the F_1_ hybrid of homozygous *Xa7/Xa7* would be developed. In the previous study, the elite *Xa7-*containing restorer lines had been developed (Huang et al. 2012; Zhang et al. 2006). In this research, we obtained the *Xa7-*containing P/TGMS line Hua1228S, and will further develop hybrid rice with the homozygous *Xa7/Xa7* genotype. Moreover, *Xa7* was susceptible to the isolates PXO99 in this study. In the next strategy, introgressing *Xa23* into Hua1228S would enhance the bacterial blight resistance spectrum of P/TGMS lines, which can be gained by another P/TGMS line Hua1015S with both *Xa23* and much of genetic background from Guangzhan63-4S, as reported previously (Jiang et al. 2015; Wang et al. 2015). In particular, it is reported that rice *Xa7* could restricts bacterial blight disease more efficiently in high temperature rice-growing seasons (Webb et al. 2010), implying the derived hybrid rice from Hua1228S would defend the bacterial blight disease more effectively in a wet and hot season in the middle-lower Yangzi River basin region of China. In short, *R* gene *Xa7* can confer high resistance to bacterial blight in P/TGMS line, and had great potential for improving the wide adaptability of two-line hybrid rice.

Recently, pyramiding of blast *R* gene has been successfully implemented in rice breeding program, thus leading to the development of the maintainer lines with broad-spectrum resistances to blast (Jiang et al. 2012). Three important *R* genes, *Pi2*, *Pi9* and *Pigm*, were recognized to confer the different resistance spectrum against blast (Deng et al. 2017; Jiang et al. 2012; Qu et al. 2006; Zhou et al. 2006). However, breeders are unable to stack these *R* genes into the same inbred line, due to allelism of *Pi2*, *Pi9* and *Pigm*. Fortunately, the breeder can transfer the different *R* genes into the male sterility line (maternal parent) and the restorer line (paternal parent), respectively, which might be a practical strategy to use the dosage effects of *Pi2*, *Pi9* and *Pigm* in hybrid rice. Hence, the newly-developed P/TGMS lines Hua1228S carrying *Pi2* should be test-crossed to the restorer lines harboring *Pi9* or *Pigm*, for the development of hybrid rice with *Pi2/Pi9* or *Pi2/Pigm* genotype. This strategy may hold great promise for efficient development of two-line hybrid rice with multiple and broad-spectrum resistances.

In conclusion, Hua1228S showed enhanced resistance in both resistance spectra and disease level of bacteria blight and blast, and it demonstrated the feasibility and usefulness of developing two-line hybrid rice with multiple and high resistance, through the improvement of P/TGMS line. Importantly, Hua1228S broadened the genetic diversity of P/TGMS line in two-line hybrid rice, and had a promising future for breeding super two-line hybrid rice with disease resistance in China. We have developed two hybrid combinations Hua Liangyou 2821 and Hua Liangyou 284 with resistance to blast and bacterial blight using Hua1228S as the female parent, which have been certificated by Hubei Province and National Committee of Crop Variety Certification and will be released for farmers in China. Such effective breeding strategies used in this study would eventually lead to the directional and accurate improvement of two-line hybrid rice.

## Methods

### Plant materials and breeding scheme

Plant materials and breeding procedure are illustrated in Fig. 1. Guangzhan63-4S was an elite *indica*-type P/TGMS line developed through the mutual cooperation by the Northern National Hybrid Rice Engineering Technology Centre and the Hefei Fengle Seed Company in China, which was used as both recurrent parent and the P/TGMS gene donor (http://www.ricedata.cn/variety/varis/601234.htm). VE6219 and YR7029–39 were two *indica*-type breeding lines developed in our lab, conferring the broad spectrum and high level resistance of rice blast and bacterial blight, respectively. Like the recurrent Guangzhan63-4S, they are adapted to rice planting area of the middle-lower Yangzi River basin region of China and have a closer relationship with Guangzhan63-4S compared with landraces. Therefore, they were used as the donor of *Pi2* and *Xa7*.

The breeding scheme is illustrated in Fig. 1. In detail, the recurrent parent Guangzhan63-4S was crossed as female parent with the donors, YR7029–39 and VE6219, in two different backcross routines. During the backcrossing process, selected individuals heterozygous at either *Xa7* or *Pi2* loci were backcrossed to Guangzhan63-4S. Though the marker-assisted selection, the plants positive for *Xa7* were selected from the BC_2_F_1_ plants derived from Guangzhan63-4S/YR7029–39, while the plants positive for *Pi2* were selected from BC_1_F_1_ plants of Guangzhan63-4S/VE6219. Subsequently, an intercross between the plants positive for *Xa7* and the plants positive for *Pi2* was carried out for stacking *Xa7* and *Pi2* in the genetic background of Guangzhan63-4S. In overall breeding procedure, marker-assisted selection was performed from generation F_1_ to MF_4_ (multiple-cross filial, MF) to screen the plants positive for *Xa7* and *Pi2*. Meanwhile, rigorous phenotypic selection for several important agronomical traits, including heading date, plant height, tiller number per plant, and pollen fertility, were conducted to select the plants with the maximal phenotypic similarity to Guangzhan63-4S, which could largely avoid linkage drag in the plants. After continuous pedigree selection combined with marker-assisted selection, a newly-developed P/TGMS line carrying both *Xa7* and *Pi2* genes was obtained, and was detected for genetic background by next-generation sequencing (NGS) technology. Finally, the desired P/TGMS line was tested for disease resistance, P/TGMS trait, and agronomic characteristics.

### Genotyping

Genomic DNA was extracted from fresh leaves of the 10-day-old seedlings following the protocol described by Murray and Thompson (1980). In the marker-assisted selection system, SSR markers RM20582 and RM527 were used to track the *Xa7* and *Pi2* genes, respectively (Chen et al. 2008; Jiang et al. 2012). Additionally, *Pi2* was confirmed using two flanking markers, RM527 and HC28 in the MF4 (Jiang et al. 2012; Jiang et al. 2012). Polymerase chain reaction (PCR) for detection of the amplified products showing small SSR variations were performed as described in the previous study (Mi et al. 2016). For the SNP variation, two photoperiod-sensitive genes *pms3* and *PMS1*, and one thermo-sensitive gene *tms5*, were analyzed by Sanger sequencing based on site-specific genomic PCR, according to previous reports (Ding et al. 2012; Fan et al. 2016; Zhou et al. 2014). Primers for marker analysis and sequencing used in this study were listed in Additional file 2: Table S1.

### Genetic background analysis based on next-generation sequencing

For genomic next-generation sequencing, the total genomic DNA of the improved P/TGMS line and original parents was extracted using the DNeasy plant mini kit (Qiagen). The library was prepared and sequenced at the Novogene Bioinformatics Institute on an Illumina Hiseq-PE150 platform. Quality control (QC), mapping, and processing of raw reads were performed as follows. High-quality reads were retained from the raw data through a series of quality control procedures, and then were aligned to the reference genome of Nipponbare (MSU7.0) by BWA software. For further analysis, we employed SAMtools and BCFtools software to detect SNP variation (Zhao et al. 2015). Homozygous SNPs of lines were extracted from vcf files, and 200,000 SNPs were used to identify genetic background of Hua1228S. To calculate genotype block across the genome, a fixed window method was used to analyze each chromosome separately with a window size of 500-kb. A pairwise distance for Hua1228S to each parent was calculated as the simple matching distance for all SNP sites in the 500-kb block using the software R 3.2 for Windows, and the block genotype was designated as the one of the three parents with highest SNP matching score. Finally, the allele frequency of donor parents was calculated as the number of block with donor genotype divided by the total block number.

### Scoring rice blast and bacterial blight resistance

All the plants were grown under the natural condition with serious blast epidemic in Lianghe village of Enshi city (N29°41′, E109°34′, 1005 m altitude), Hubei province, China. These plants were scored for leaf blast at tillering, disease incidence percentage and loss rate of infection on the neck of the rice panicle at maturity stage according to the methods and standards described by Ni et al. (2015). In order to detect the resistance spectrum of rice blast, 33 isolates of *M. oryzae* collected from Guangdong provinces of China were used for artificial inoculation of leaf blast at the seedling stage in greenhouse conditions. Disease reaction was evaluated using a 0 to 9 rating system as described by Wang et al. (2013), where 0 to 3 is resistant and 4 to 9 is susceptible. The rice variety CO39 was used as a susceptible control.

Seven strains of *Xoo* were applied for artificial inoculation of bacterial blight separately in the experimental field of Huazhong Agricultural University (N30°35′, E114°17′, 16 m altitude) according to the leaf-clipping method as described by Jiang et al. (2015). All plants were inoculated at growth stage of maximum tillering. Of the seven races, ZHE173 and GD1358 caused severe bacterial blight epidemics in the rice-growing regions of southern China (Jiang et al. 2015). PXO61, PXO86, PXO79 and PXO99 were collected from the Philippines, and T1 from Japan (Kihupi et al. 2001). Bacterial suspension was prepared following the method described previously by Maruthasalam et al. (2007). Disease symptoms were recorded by measuring the lesion length three weeks after inoculation (Jiang et al. 2015).

### Fertility-sterility alteration test in the field

In 2013, one hundred and twenty seeds of each line were sown every 15 days from April 1st to July 1st at the experimental farm of Huazhong Agricultural University, Wuhan city, Hubei province, China. Forty uniform and healthy rice seedlings at the five-leaf stage were transplanted in fields at a planting density of 20.0 cm between plants in a row, and 25.0 cm apart between rows. At the heading stage, pollen grains of the top five florets of primary panicles were mixed and stained with 1% I_2_-KI solution. Five plants of each line were investigated, as described by Mi et al. (2016). The daily mean temperature data were provided by the Agricultural Meteorology Department of Huazhong Agricultural University. The dynamic pollen fertility of Hua1228S and Guangzhan63-4S was observed with two-day intervals from July 12th to September 26th in 2013.

### Agronomic performance test

Agronomic traits of the newly-developed P/TGMS line and Guangzhan63-4S were evaluated under the natural field, during the summer season of 2014 at the experimental farm of Huazhong Agricultural University, Wuhan city, Hubei province, China and the spring season of 2015 at the Rice Breeding Station of Huazhong Agricultural University, Lingshui county (N18°30′, E110°01′, 10 m altitude), Hainan province, China. Each line was planted in a plot with four sowing dates in 2014, namely April 15th, May 5th, May 25th, and June 15th, and three sowing dates in 2015, namely November 20th, November 30th, and December 10th. Each plot consisted of five rows with 10 plants per row at a spacing of 16.7 cm × 26.7 cm. Field management followed the normal agricultural practices. The heading date of each line was recorded according to the method described previously (Mi et al. 2016). At maturity, five plants in the middle of the central row in each plot were taken and measured for agronomic traits as described by Jiang et al. (2015).

### Evaluation of derived hybrids in Rice variety region trial

The performance evaluation of hybrid combinations, including blast resistance, bacterial blight resistance and yield, was conducted by Hubei Province and National Committee of Crop Variety Certification in both 2015 and 2016, according to methods and standards of rice variety region trial (http://www.srvt.net).

### Statistical analysis

Statistical analysis was conducted by the software SPSS statistics 17.0 for Windows (IBM, Armonk, NY, USA). The two-tailed *t*-test was used for comparing agronomic traits of the improved P/TGMS line Hua1228S with that of the control line Guangzhan63-4S. It is also used for comparing the disease resistance and yield traits of the hybrid combinations derived from Hua1228S with that of the control variety Feng Liangyou 4.

## Additional files

Additional file 1: **Table S2.** Genotype of three P/TGMS genes by PCR-based sequencing. (XLSX 10 kb)

Additional file 2: **Table S1.** Primers sequence used in this study. (XLSX 10 kb)

## Abbreviations

P/TGMS: Photoperiod- and thermo-sensitive male sterile
*R*: Resistance
*Xoo*: *Xanthomonas oryzae* pv. *Oryzae*
